# Revisiting Anti-tuberculosis Activity of Pyrazinamide in Mice

**DOI:** 10.4172/2161-1068.1000145

**Published:** 2014-05-05

**Authors:** Deepak V. Almeida, Sandeep Tyagi, Si-Yang Li, Kristina Wallengren, Alexander S. Pym, Nicole C. Ammerman, William R. Bishai, Jacques H. Grosset

**Affiliations:** 1 Center for Tuberculosis Research, Johns Hopkins University School of Medicine, Baltimore, Maryland, USA; 2 KwaZulu-Natal Research Institute for Tuberculosis and HIV (K-RITH), Durban, South Africa

## Abstract

The mechanism of action of pyrazinamide, a key sterilizing drug in the treatment of tuberculosis, remains elusive; pyrazinamide is a pro-drug that requires activation by a bacterial-encoded enzyme, and its activity is most apparent on non-replicating *Mycobacterium tuberculosis*. Recently, it has been suggested that pyrazinamide might exert also some host-directed effect in addition to its antimicrobial activity. To address this possibility, three sequential experiments were conducted in immune-competent BALB/c and in immune-deficient, athymic nude mice. In the first experiment, BALB/c mice infected with *M. bovis*, which is naturally resistant to pyrazinamide because it is unable to activate the drug, were treated with standard drug regimens with and without pyrazinamide to specifically detect a host-directed effect. As no effect was observed, pyrazinamide activity was compared in *M. tuberculosis*-infected BALB/c and nude mice to determine whether the effect of pyrazinamide would be reduced in the immune deficient mice. As pyrazinamide did not appear to have any affect in the nude mice, a third experiment was performed in which rifampin was replaced with rifapentine (a similar drug with a longer half-life) to permanently suppress mycobacterial growth. In these experimental conditions, the antimicrobial effect of pyrazinamide was clear. Therefore, the results of our studies rule out a significant host-directed effect of pyrazinamide in the TB infected host.

## Introduction

Although it is the most sterilizing drug in the armamentarium against tuberculosis (TB), an indispensable component of first-line drug combination for drug-susceptible TB [1] and part of all recommended drug regimens for multidrug-resistant TB [2,3], pyrazinamide (PZA) remains a compound with no well-defined target of action. It is a pro-drug that needs to be activated by the mycobacterial enzyme nicotinamidase, named pyrazinamidase (PZase) [4], to its active form, pyrazinoic acid (POA). After its activation, it is generally accepted [5] that POA is expelled from the mycobacterial cell by an efflux pump and is protonated in the extracellular environment. Upon re-entry into the bacterial cell, this proton is released, decreasing the cytoplasmic pH and thus causing lethal damage through several possible mechanisms, including membrane disruption [5,6], inhibition of fatty acid synthetase [7] and inhibition of trans-translation [8] of *Mycobacterium tuberculosis*. In both human and mouse TB chemotherapy, the sterilizing activity of PZA becomes evident when the bacteria are not actively replicating [9–12], likely because the PZA-induced damage should accumulate to become lethal.

PZase is encoded by the 561-nucleotide *pncA* gene [5,13]. Mutations in *pncA* can result in lost or reduced PZase activity, and such mutations, of which there is a high degree of diversity [14], have been associated with PZA resistance in clinical isolates of *M. tuberculosis*, as well as in *M. bovis*, which is naturally resistant to the drug [13,15]. Mutations in *pncA* are considered to be the primary mechanism of PZA resistance in *M. tuberculosis* [5,16]; however, mutations in this gene are only associated with approximately 70% of observed PZA resistance [17–19]. The search for other mechanisms of PZA resistance has resulted in the identification of mutations in *rpsA*, encoding the ribosomal protein S1 (RpsA, Rv1630), a vital protein involved in trans-translation [8], and, more recently, in panD, which encodes an aspartate decarboxylase involved in synthesis of β-alanine, a precursor for pantothenate and co-enzyme A biosynthesis [20]. In addition to PZA resistance associated with *pncA, rpsA* or *panD* mutations, it has also been reported that resistance can result from changes in *pncA* expression, altered PZA update, or dysregulated POA efflux [21].

PZA is an analog of nicotinamide, a compound long known as having some antituberculosis activity in the mouse model [22,23]. Nicotinamide is the amide of nicotinic acid (vitamin B_3_, niacin), and its observed anti-TB activity was thought to be due to its role as a vitamin (thus providing a host-directed effect) rather than anti-mycobacterial, a concept that was supported when nicotinamide was found to be beneficial in the treatment of a variety of inflammatory skin disorders [24]. Furthermore, nicotinamide can be converted to its acid form by the *M. tuberculosis* PZAse [4], and nicotinic acid has well-described lipid-lowering and anti-inflammatory properties [25]. In 2009, Mendez and colleagues reported that PZA had anti-leishmanial activity in vitro and in C57BL/6 mice, and interestingly also demonstrated the addition of PZA to uninfected macrophages lead to activation of the cells and release of pro-inflammatory cytokines [26]. These findings, taken together with similarities between PZA and nicotinamide, suggest that PZA may have host-directed activity in addition to its antimicrobial properties.

In order to evaluate the possibility of some host-directed activity of PZA in TB treatment, a series of three experiments were performed in immune-competent and immune-deficient mouse models of TB. The results clearly demonstrate the antibacterial potency of PZA associated with PZAse activity, but cannot demonstrate significant host-directed effect of this drug.

## Materials and Methods

### Antimicrobials

Isoniazid (INH), ethambutol (EMB) and rifampin (RIF) were purchased from Sigma, and PZA was purchased from Fisher Scientific International. Moxifloxacin (MXF) and rifapentine (RFP) were donated from Bayer and Sanofi-Aventis Pharmaceuticals, respectively. All stock solutions were prepared in distilled water except for RPT for which tablets (PRIFTIN containing 150 mg of the active ingredient RPT per tablet) were ground into a fine powder, suspended in 0.05% (wt/vol) agarose and briefly sonicated before use. Drug solutions were prepared weekly and stored at 4°C.

### Bacterial strains

*M. tuberculosis* strain H37Rv (ATCC^®^ 27294^™^) [27] and *M. bovis* strain Ravenel (ATCC^®^ 35720^™^) [28] were passaged in mice, frozen in 1 ml aliquots and stored at −80°C. For each infection, an aliquot was thawed and sub-cultured in Middlebrook 7H9 broth (Gibco) supplemented with 10% (vol/vol) oleic acid-albumin-dextrose-catalase (Difco) and 0.05% (vol/vol) Tween-80 (Sigma).

### Mice and aerosol infection

Six-week-old female BALB/c mice and congenitally athymic nu/nu (nude) [29,30] outbred Swiss mice were purchased from Charles River. In all three experiments, mice were infected by the aerosol route using the inhalation exposure system (Glascol, Inc.) with a log-phase culture at an optical density at 600 nm of approximately 1.0 to achieve an implant of approximately 5×10^3^ CFU in the lungs. After infection, mice were randomized into treatment groups. All animal procedures were approved by the Institutional Animal Care and Use Committee of the Johns Hopkins University School of Medicine.

### Drug administration

All antimicrobials were administered orally (by gavage) using an esophageal cannula. Drugs were administered once daily, 5 days per week, in a total volume of 0.2 ml. In all three experiments, treatment was initiated 14 days after infection (Day 0), and the drugs were administered at the following daily doses: 10 mg/kg for INH, RIF and RPT; 100 mg/kg for EMB and MXF; and 150 mg/kg for PZA. The doses were selected to match the area under the concentration curve values obtained with the recommended human dosages [9,31–33]. RIF was administered one hour prior to the administration of the other drugs to avoid an adverse pharmacokinetic interaction [31,34].

### Assessment of treatment efficacy

Treatment efficacy as assessed on the basis of lung CFU counts. Untreated mice were routinely sacrificed (i) on the day after infection (Day −13) to determine the numbers of CFU implanted in the lungs and (ii) at treatment initiation (Day 0) to determine the pre-treatment CFU count. To assess antimicrobial activity, five mice from each treatment group were sacrificed at regular intervals to determine the reduction in lung CFU counts. Lungs were removed under aseptic conditions, placed in sterile phosphate-buffered-saline, homogenized and plated on selective 7H11 agar (Becton-Dickinson) as previously described [33,35]. Plates were incubated for 4 weeks at 37°C with 5% CO_2_ before CFU counts were determined.

### Statistical analysis

CFU counts were log_10_ transformed before analysis. Group means or differences in means for experimental treatment groups were compared with the control group by one-way analysis of variance with Dunnett’s post-test. Group proportions were compared using Fisher’s exact test and adjusting for multiple comparisons. All analyses were performed with GraphPad Prism version 6.0.

## Results

### Experiment 1

We hypothesized that if PZA has host-directed activity (in addition to its antimicrobial activity), then we should be able to specifically observe this activity in mice infected with a PZA-resistant organism such as *M. bovis*. Our first experiment addressed this issue by comparing the activity of RIF, INH and RIF+INH with or without PZA in *M. bovis*-infected BALB/c mice (see experimental scheme presented in Table 1). Mice were infected by aerosol with *M. bovis* strain Ravenel, and the day after infection (Day −13), the mean lung log_10_ CFU count (standard deviation [SD]) was 3.73 (0.13). After two weeks (at the time of treatment initiation), the mean log_10_ CFU counts had increased to 6.56 (0.12). After Week 1, mice started dying in the untreated control group, and the surviving mice maintained high CFU counts (greater than 8 log_10_), indicating that the *M. bovis* strain was fully virulent.

During the 16 weeks of treatment, mice receiving PZA alone were similar to the untreated control group for CFU counts and mortality (Table 2), demonstrating a total lack of PZA activity. All mice treated with RIF, INH or RIF+INH responded well to the treatment, with the decline in CFU counts, as usual, steeper in mice treated with INH alone than in mice treated with RIF alone, and in the mice treated with the INH+RIF combination than in mice treated with INH alone. The key finding was that the addition of PZA to any of these drug regimens did not influence the decline in CFU counts either negatively or positively, indicating a lack of additive, synergistic or antagonistic effect of PZA, either microbe- or host-directed.

### Experiment 2

Based on the results of the first experiment, we considered that PZA may need to be converted to its acid form, POA (a niacin analogue), in order to exert a host-directed effect, and thus assessment of any host-directed activity would require using a mycobacterial strain with a functional PZase. Furthermore, we hypothesized that any host-directed activity, but not the antimicrobial activity, of PZA could be altered in immune-deficient mice compared to immune-competent mice. Therefore, for the second experiment, we assessed PZA-specific activity using *M. tuberculosis* strain H37Rv (wild-type *pncA* and fully PZA-susceptible) to infect both immune-competent BALB/c mice and immune-deficient athymic nude mice. As shown in the experimental scheme presented in Table 3, our primary comparison was between mice treated with RIF+EMB with mice treated with RIF+EMB+PZA in both mouse strains.

The day after aerosol infection (Day −13), the mean lung log_10_ CFU count (SD) was 3.80 (0.09) in BALB/c mice and 3.90 (0.14) in the nude mice. Two weeks later, at the start of treatment (Day 0), the mean log_10_ CFU counts had increased to 8.01 (0.04) and 8.61 (0.44) in the BALB/c and nude mice, respectively, with a single nude mouse having a CFU count as high as 9.3 log_10_ CFU; the difference in CFU counts between the two mouse strains was statistically significant (p=0.01). All remaining untreated BALB/c and nude mice were dead or moribund by the fourth week after infection.

**Figure 1:**
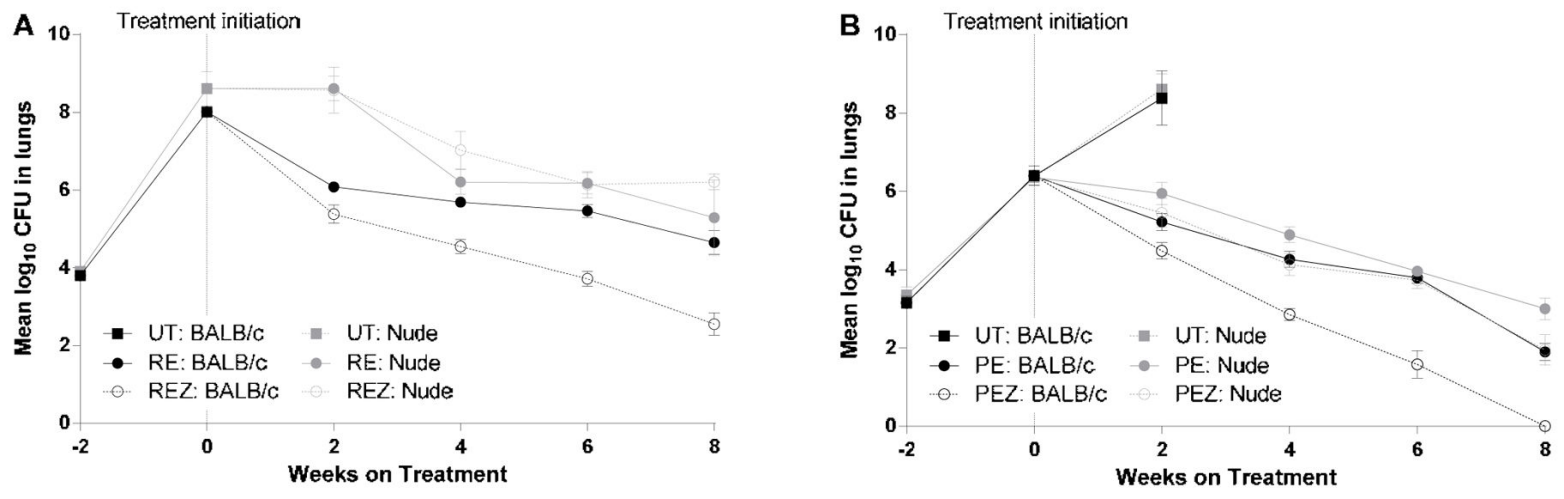
The contribution of PZA to RIF- and RPT- containing regimens in *M. tuberculosis*-infected BALB/c and nude mice. (A) Mean log_10_ CFU counts in the lungs of mice receiving RIF+EMB with or without PZA. (B) Mean log_10_ CFU counts in the lungs of mice receiving RPT+EMB with or without PZA. Treatment was initiated on Day 0 (14 days after infection), and mice were treated 5 days per week at the following doses: RIF (R), 10 mg/kg; EMB (E), 100 mg/kg; PZA (Z), 150 mg/kg; RPT (P), 10 mg/kg. Error bars represent standard deviation. UT, untreated.

In mice treated with the standard regimen (RIF+INH+PZA+EMB), the lung log_10_ CFU counts steadily declined in both the BALB/c and nude mice to reach 3.09 (0.14) and 3.84 (0.17), respectively, by Week 8 (Table 4). The overall reduction of the log_10_ CFU counts with the standard control regimen (RIF+INH+PZA+EMB) was 4.92 and 4.77 in the BALB/c and nude mice, respectively (p>0.05), indicating that the response to this regimen was similar in the immune-competent and immune-deficient mice. However, the findings were totally different in mice treated with RIF+EMB and RIF+EMB+PZA (Figure 1). In BALB/c mice, the PZA-containing regimen was much more potent than RIF+EMB; the lung log_10_ CFU counts were 2.55 (0.29) and 4.65 (0.31) after 8 weeks of the regimens with or without PZA, respectively. Conversely, in the nude mice, the lung log_10_ CFU counts after 8 weeks of treatment were 5.29 (0.97) for RIF+EMB and 6.21 (0.20) for RIF+EMB+PZA. The benefit of adding PZA was clearly observed in the BALB/c mice but was nil in the nude mice.

### Experiment 3

In *M. tuberculosis*-infected nude mice, the bacilli continue to actively multiply until the mice succumb to the infection, *i.e.*, a non-replicating, chronic infection state is not established [36]. Because PZA is known to be active only against non-replicating bacilli, it is possible that the lack of PZA activity observed in the nude mice in our second experiment was due to the lack of permanent suppression of bacterial multiplication by the other administered drugs. Therefore, we considered that replacing RIF which has a steady-state half-life in mice of approximately 2 hours, with RPT, which has a steady state half-life of approximately 14 hours in mice [37], may allow for continuous suppression of bacterial replication, thus allowing PZA activity to be observed. Based on this, a third experiment was conducted in which the specific contribution of PZA to RPT-containing regimens was assessed in both BALB/c and nude mice (see experimental scheme presented in Table 5).

The day after infection (Day −13), the mean lung log_10_ CFU count (SD) was 3.26 (0.16) and 3.35 (0.21) in the BALB/c and nude mice, respectively. By the initiation of treatment on Day 0, the mean log_10_ CFU counts had increased to 6.40 (0.25) in the BALB/c mice and 6.36 (0.20) in the nude mice. By 4 weeks after infection, the log_10_ CFU counts had reached 8.38 (0.69) and 8.61 (0.39) in the untreated control BALB/c and nude mice, respectively, and by 6 weeks post-infection, all control mice died.

The addition of PZA to RPT+EMB, RPT+INH+EMB and PRT+MXF+EMB was beneficial in both the BALB/c and nude mice (Table 6), indicating that PZA was active in the immune-deficient mice when the bacillary growth had been totally suppressed by the administration of RPT. However, it should be noted that the responses to all of the PZA-containing regimens as well as to all of the PZA-free regimens were more limited in the nude mice compared to the BALB/c mice, likely because the latter miss the positive contribution of natural and specific immunity.

## Discussion

The reported experiments were designed to address the issue of whether PZA has a host-directed effect in addition to its antimicrobial effect. Clearly, even in the immune-competent BALB/c host, we confirmed the well-known notion that PZA alone or in combination with RIF and/or INH has no activity at all in mice infected with *M. bovis* [4], ruling out any host-directed activity of PZA in these experimental conditions (Table 2). However, as a host-directed effect might result from POA activity, and as *M. bovis* cannot activate PZA into POA because of its natural mutation in the *pncA* gene, the inactivity of PZA in *M. bovis*-infected BALB/c mice did not exclude the possibility that activated PZA (*i.e*., POA) may have such activity.

Therefore, we next assesseded the activity of PZA in mice infected with the PZA-activating *M. tuberculosis* strain H37Rv and performed the assessment in both immune-competent BALB/c and immune-deficient nude mice expecting that PZA in the nude mice lacking activated T cells would only express antimicrobial effect and no host-related immune effect. Actually the addition of PZA to the RIF+EMB combination was not beneficial at all in nude mice, while it strongly potentiated the antimicrobial activity in the immune competent BALB/c mice (Table 4) suggesting that the host-directed immune effect was playing a major role because PZA was not active in nude mice. However, we speculated that in the nude mice, the co-administered drugs RIF+EMB may not have been able to totally suppress bacterial replication, which is possible in the BALB/c mice when the activity of the drugs is also supported by a functional immune system. Considering the short half-life of RIF in mice (approximately 2 hours during steady-state [37], it was possible that the infected nude mice receiving RIF+EMB had windows of time each day when bacterial multiplication was not completely suppressed and therefore were not permitting PZA to express its antimicrobial activity.

A third experiment was then conducted in which the bacillary growth was permanently suppressed by the daily use of the long-lasting RPT instead of RIF. In these experimental conditions, the benefit of adding PZA was clearly evidenced confirming the direct antimicrobial activity of PZA and infirming the host-related effect.

Furthermore it is interesting to note that the antimicrobial activity of all PZA-free and PZA-containing regimens, was significantly inferior in nude compared to the BALB/c mice. The immune status of the host is therefore an important positive factor in the response to antibiotic treatment. In conclusion, our studies did not confirm the existence of a significant host-directed effect of PZA in TB therapy. It is however possible that some host-directed effect of PZA could be observed in some experimental conditions. If that was the case, it remains to determine by which mechanism a host-directed activity of PZA might be expressed. It is possible that *Leishmania*, like *M. tuberculosis*, through a nicotinamidase encoded by a *pncA* gene [38], can produce on treatment with PZA large amounts of POA which act directly or indirectly through a receptor to mediate an immune response. Recently, Manca and colleagues reported that administration of PZA to *M. tuberculosis*-infected human monocytes and mice alters the host immune response in an anti-inflammatory nature [39]. But based on our work, it seems that any host-directed effect of PZA is likely to be modest and therefore difficult to be evidenced in the presence of other antimicrobial drugs and a robust host immune response.

## Figures and Tables

**Table 1:** Scheme for experiment 1: assessment of PZA activity in *M. bovis*-infected mice.

	No. of mice to be sacrificed at the following time points							
	Day −13	Day 0	Wk 1	Wk 2	Wk 4	Wk 8	Wk 16	Total
**Control Regimens**								
Untreated	10	10	5	5	5	5	5	45
RIF			5	5	5	5	5	25
INH			5	5	5	5	5	25
PZA			5	5	5	5	5	25
RIF+INH			5	5	5	5	5	25
**Test Regimens**								
RIF+PZA			5	5	5	5	5	25
INH+PZA			5	5	5	5	5	25
RIF+INH+PZA			5	5	5	5	5	25
Total Mice	10	10	40	40	40	40	40	220

Treatment was initiated two weeks after infection at Day 0 and was administered for 16 weeks.

**Table 2:** Lung CFU counts in *M. bovis*-infected mice treated with different drug regimens with and without PZA (Experiment 1).

	Mean (SD) log_10_ *M. bovis* CFU counts in mouse lungs at the following time points						
	Day −13	Day 0	Wk 1	Wk 2	Wk 4	Wk 8	Wk 16
**Control Regimens**							
Untreated	3.73 (0.13)	6.56 (0.12)	7.67 (0.17)	8.69 (0.71)	8.11 (0.33)	8.79 (0.49)*	
RIF			7.15 (0.30)	6.87 (0.29)	6.75 (0.08)	6.09 (0.16)	5.79 (0.26)
INH			6.46 (0.11)	6.31 (0.22)	5.70 (0.16)	4.69 (0.31)	3.86 (0.20)
PZA			7.75 (0.21)	8.17 (0.17)	8.31 (0.62)	7.5**	
RIF+INH			5.71 (0.19)	5.03 (0.05)	4.40 (0.18)	2.72 (0.14)	0
**Test Regimens**							
RIF+PZA			7.04 (0.19)	7.27 (0.51)	6.96 (0.08)	5.94 (0.23)	5.82 (0.19)
INH+PZA			6.99 (0.52)	6.38 (0.36)	5.80 (0.23)	5.19 (0.36)	4.12 (0.74)
RIF+INH+PZA			5.47 (0.24)	5.09 (0.28)	4.39 (0.14)	2.63 (0.23)	0.68 (1.20)

Treatment was initiated two weeks after infection at Day 0 and was administered for 16 weeks. Drugs were administered 5 days per week at the following doses: RIF, 10 mg/kg; INH, 10 mg/kg; PZA, 150 mg/kg.

* CFU counts from two surviving mice.

** CFU counts from a single mouse that survived to this time point.

**Table 3:** Scheme for experiment 2: assessment of PZA activity in *M. tuberculosis-*infected BALB/c and nude mice.

	No. of mice to be sacrificed at the following time points						
	Day −13	Day 0	Wk 2	Wk 4	Wk 6	Wk 8	Total
**Control Regimens**							
Untreated	5	5	5				15
RIF+INH+PZA+EMB			5	5	5	5	20
RIF+EMB			5	5	5	5	20
**Test Regimen**							
RIF+EMB+PZA			5	5	5	5	20
Total Mice	5	5	20	15	15	15	75

Treatment was initiated two weeks after infection at Day 0 and was administered for 8 weeks. Ethambutol was included to prevent the emergence of drug resistance in the nude mice. The same scheme was used for 75 BALB/c and 75 nude mice

**Table 4:** Lung CFU counts in *M. tuberculosis*-infected BALB/c and nude mice treated with RIF+EMB with or without PZA (Experiment 2).

	Mouse strain	Mean (SD) log10 CFU counts in mouse lungs at the following time points					
		Day −13	Day 0	Wk 2	Wk 4	Wk 6	Wk 8
**Control Regimens**							
Untreated	BALB/c	3.80 (0.11)	8.01 (0.05)				
	Nude	3.90 (0.14)	8.61 (0.44)				
RIF+EMB+PZA+INH	BALB/c			5.73 (0.30)	5.22 (0.06)	4.10 (0.19)	3.09 (0.14)
	Nude			6.19 (0.13)	6.37 (0.41)	4.71 (0.39)	3.84 (0.17)
RIF+EMB	BALB/c			6.08 (0.13)	5.69 (0.03)	5.46 (0.17)	4.65 (0.31)
	Nude			8.61 (0.31)	6.21 (0.32)	6.17 (0.27)	5.29 (0.97)
**Test Regimen**							
RIF+EMB+PZA	BALB/c			5.38 (0.23)	4.55 (0.18)	3.72 (0.19)	2.55 (0.29)
	Nude			8.57 (0.59)	7.03 (0.48)	6.14 (0.34)	6.21 (0.20)

Treatment was initiated two weeks after infection at Day 0 and was administered for 8 weeks. Drugs were administered 5 days per week at the following doses: RIF, 10 mg/kg; EMB, 100 mg/kg; PZA, 150 mg/kg; INH, 10 mg/kg

**Table 5:** Scheme for experiment 3: assessment of PZA activity in *M. tuberculosis-*infected BALB/c and nude mice treated with RPT instead of RIF.

	No. of mice to be sacrificed at the following time points						
	Day −13	Day 0	Wk 2	Wk 4	Wk 6	Wk 8	Total
**Control Regimens**							
Untreated	5	5	5				15
RPT+INH+EMB			5	5	5	5	20
RPT+MXF+EMB			5	5	5	5	20
RPT+EMB			5	5	5	5	20
**Test Regimens**							
RPT+INH+EMB+PZA			5	5	5	5	20
RPT+MXF+EMB+PZA			5	5	5	5	20
RPT+EMB+PZA			5	5	5	5	20
Total Mice	5	5	30	30	30	30	135

Treatment was initiated two weeks after infection at Day 0 and was administered for 8 weeks. Five untreated mice were kept after Week 2 for mortality assessment. The same scheme was used for 140 BALB/c and 140 nude mice

**Table 6:** Lung CFU counts in *M. tuberculosis*-infected BALB/c and nude mice treated with RPT-containing regimens with or without PZA (Experiment 3).

	Mouse strain	Mean (SD) log_10_ CFU counts in mouse lungs at the following time points					
		Day −13	Day 0	Wk 2	Wk 4	Wk 6	Wk 8
**Control Regimens**							
Untreated	BALB/c	3.16 (0.16)	6.40 (0.25)	8.38 (0.69)			
	Nude	3.35 (0.21)	6.36 (0.20)	8.61 (0.39)			
RPT+INH+EMB	BALB/c			5.32 (0.25)	4.06 (0.14)	3.05 (0.31)	1.10 (0.08)
	Nude			5.78 (0.13)	4.90 (0.63)	3.84 (0.10)	3.08 (0.35)
RPT+MXF+EMB	BALB/c			5.18 (0.21)	4.16 (0.11)	3.57 (0.16)	2.02 (0.17)
	Nude			5.70 (0.43)	4.53 (0.45)	3.91 (0.30)	2.77 (0.55)
RPT+EMB	BALB/c			5.22 (0.22)	4.26 (0.20)	3.79 (0.14)	1.90 (0.22)
	Nude			5.95 (0.28)	4.89 (0.20)	3.96 (0.04)	3.00 (0.28)
**Test Regimen**							
RPT+INH+EMB+PZA	BALB/c			5.22 (0.35)	3.48 (0.30)	2.85 (0.22)	0.43 (0.50)
	Nude			5.72 (0.28)	4.63 (0.35)	3.60 (0.44)	2.55 (0.40)
RPT+MXF+EMB+PZA	BALB/c			3.93 (0.52)	2.75 (0.10)	1.17 (0.50)	0
	Nude			5.23 (0.24)	3.90 (0.32)	3.67 (0.33)	0.98 (0.39)
RPT+EMB+PZA	BALB/c			4.48 (0.21)	2.85 (0.15)	1.58 (0.35)	0
	Nude			5.45 (0.37)	4.13 (0.28)	3.73 (0.22)	1.95 (0.39)

Treatment was initiated two weeks after infection at Day 0 and was administered for 8 weeks. Drugs were administered 5 days per week at the following doses: RPT, 10 mg/kg; INH, 10 mg/kg; EMB, 100 mg/kg; MXF, 100 mg/kg; PZA, 150 mg/kg
